# Combating antimicrobial resistance in Africa: a strategic roadmap for surveillance, stewardship, and research

**DOI:** 10.3389/fcimb.2025.1714021

**Published:** 2025-12-17

**Authors:** Ifeanyi Elibe Mba, Favour Temiloluwa Martins

**Affiliations:** 1 Department of Pharmaceutical Microbiology, Faculty of Pharmacy, University of Ibadan, Ibadan, Oyo, Nigeria; 2 Department of Biological Sciences, Faculty of Sciences, Lead City University, Ibadan, Nigeria

**Keywords:** Africa, AMR surveillance, antimicrobial resistance (AMR), antimicrobial stewardship, clinical research capacity, health system strengthening, low- and middle-income countries (LMICs)

## Abstract

Africa stands at a pivotal moment in public health: the continent faces a high and growing burden of infectious diseases driven in part by antimicrobial resistance (AMR) yet remains underrepresented in global clinical research and surveillance initiatives. Despite advances in technology, infrastructure and human capital in some regions, persistent gaps in laboratory capacity, epidemiologic surveillance, data systems, governance, funding and clinical research impede timely detection, containment and evidence-based management of drug-resistant infections. This review synthesizes the current landscape of AMR surveillance and clinical research capacity across African countries, highlighting the major structural and often over-looked barriers and also exemplary local initiatives that demonstrate scalable progress. We argue that linking strengthened AMR surveillance with concerted investment in clinical research, laboratory networks, antimicrobial stewardship, data governance and policy reforms will enable a shift from reactive responses to proactive, system-wide AMR prevention and containment strategies. Drawing on published literature, policy documents and regional case studies, this review proposed a practical, phased roadmap for integrated surveillance-led healthcare reform tailored to African contexts and realities, with concrete recommendations. It also defined actionable priorities and framework to accelerate AMR detection, research, and containment across Africa, ultimately reducing the burden of drug-resistant infections and strengthening health system resilience in Africa.

## Introduction

Antimicrobial resistance (AMR) has emerged as one of the greatest global public health threats of the 21st century (Tang et al., 2023; Sharma et al., 2024). AMR occurs when bacteria are no longer responsive to antibiotics intended to inhibit their growth or kill them (Uddin et al., 2021). It is fundamentally an evolutionary phenomenon, as pathogens naturally acquire resistance over time. However, human activities have greatly accelerated this process. Key drivers of AMR include the misuse of antibiotics in humans and animals, poor healthcare practices, and environmental factors (Akpoji et al., 2022; Bottery et al., 2021). Inadequate infection prevention and control (IPC), poor water, sanitation, and hygiene (WASH), and weak healthcare infrastructure facilitate the transmission of resistant organisms. Substandard or counterfeit antibiotics, limited access to diagnostics and vaccines, and insufficient regulation or enforcement further compound the problem (Kelesidis and Falagas, 2015; Sharma et al., 2024; Olagunju et al., 2025). Socioeconomic factors such as poverty, overcrowding, and lack of education also promote inappropriate antibiotic use and limit the capacity to respond effectively to AMR. These challenges are especially acute in many African nations, where health systems are often under-resourced. Hospitals face overcrowding and water scarcity, laboratory capacity is constrained, and antibiotics are frequently obtained without a prescription. All these conditions have created a perfect storm for the emergence and spread of resistance on the continent. Additionally, some organisms are inherently resistant to antibiotics, even without prior exposure (Akpoji et al., 2022; Bottery et al., 2021).

AMR poses a critical public health threat, especially in Africa, where the burden is disproportionately high, with serious health and economic consequences (Sulis et al., 2022). In 2019 alone, the region recorded approximately 27.3 AMR-related deaths per 100,000 population, compared to 6.3 per 100,000 in high-income countries (Kariuki et al., 2022; Africa CDC, 2024). Projections suggest that by 2050, AMR could cause over 10 million deaths annually and cost the global economy more than $100 trillion if left unaddressed (Akpoji et al., 2022; Littmann and Simonsen, 2019). Despite its burden and impact, Africa lacks robust surveillance systems to effectively tackle the crisis. Controlling AMR requires context-specific, integrated frameworks built on strong governance, local leadership, and sustainable financing. Surveillance is foundational, as it enables the detection of emerging resistance trends and informs targeted interventions (Perez and Villegas, 2015). However, Africa faces challenges such as weak laboratory systems, limited diagnostic capabilities, and inadequate funding necessary for effective surveillance.

While there are efforts in various sectors to mitigate these challenges, they remain limited. For example, the Global Antibiotic Resistance Partnership has been established in only a few African countries (Anyaegbunam et al., 2023). Even at the healthcare facility level, many African nations face challenges in implementing antimicrobial stewardship programs. Medical interventions and public health measures are often direct or indirect outcomes of clinical research. However, there remains a significant gap in clinical research across Africa — a continent heavily burdened by AMR. Currently, only 2–4% of global clinical trials are conducted in African countries or involve African participants (Saleh et al., 2024; Ndembi et al., 2024). Moreover, as of today, only four African nations have fulfilled the World Health Organization’s 2001 commitment to allocate at least 15% of their GDP to health and science (Arize et al., 2024; Chipunza and Ntsalaze, 2024). Yet, the return on investment in health research and community well-being is substantial — estimated at nearly 20 to 1 (Turner et al., 2023; Okoye et al., 2022). Although many other factors contribute to this gap, health system weaknesses, brain drain, and poor infrastructure are the major barriers to scientific progress. In fact, to date, many health facilities across the continent remain overcrowded and under-resourced, lacking essential diagnostic tools and medical equipment (Oleribe et al., 2019; Azevedo, 2017). Alarmingly, about 62% of patients in Africa lack access to routine diagnostics (Azevedo, 2017), making it extremely difficult — though not impossible — to conduct research that could significantly improve healthcare outcomes.

Overall, effective AMR surveillance and clinical research frameworks are crucial to combating the growing threat of drug-resistant infections and ensuring equitable access to quality healthcare across all levels of society. Until these issues are addressed, the gap between Africa and other continents will remain wide. This review synthesizes the current landscape of AMR surveillance and clinical research capacity across African countries, highlighting major structural and often overlooked barriers, as well as exemplary local initiatives that demonstrate scalable progress in Africa. It examines the current state of AMR surveillance and clinical research capacity across Africa, with a focus on how system gaps affect timely diagnosis of drug-resistant infections. It also assesses how integrated surveillance, governance reforms, and regional initiatives can strengthen prevention and containment of AMR at population level. By synthesizing literature, policy documents, and case studies, this work outlines a practical roadmap for surveillance-led health system strengthening tailored to African contexts. Ultimately, this review defines actionable priorities and frameworks to accelerate AMR detection, clinical research, and the containment of infectious superbugs across Africa, with the aim of reducing the burden of drug-resistant infections and strengthening health system resilience across the continent.

### Multifaceted impact of antimicrobial stewardship programs – lessons and the way forward

Antimicrobial Stewardship Programs (ASPs) are defined as coordinated interventions designed to promote the appropriate use of antimicrobial agents by optimizing prescribing practices (Khadse et al., 2023). These programs are multifaceted and typically involve a multidisciplinary team of infectious disease physicians, pharmacists, microbiologists, and infection prevention experts. The primary objectives of ASPs are to enhance patient care, improve clinical outcomes, and reduce adverse effects. A core objective of ASPs is to optimize antimicrobial utilization. The effectiveness of these interventions is frequently measured using specific metrics, the most common being Defined Daily Dose (DDD) or Days of Therapy (DOT) per 1,000 patient-days (Antimicrobial Stewardship Metrics, 2025). The Defined Daily Dose, developed by the World Health Organization (WHO), represents the assumed average daily dose of a drug used for its main indication in adults. In contrast, Days of Therapy measures the actual number of days a patient receives an antimicrobial and has become the preferred metric due to its capacity to track the true duration of therapy.

The body of evidence consistently and strongly demonstrates that ASPs are highly effective in reducing antimicrobial consumption (Antimicrobial Stewardship Programs, 2025). A systematic review and meta-analysis of 52 studies concluded that ASPs were associated with a pooled reduction in total antibiotic consumption by 19% (Ya et al., 2023). For more restricted antimicrobial classes, the impact was even more pronounced, with reductions of up to 27% observed in hospital settings. This reduction is achieved through a range of targeted interventions that improve the appropriateness of prescriptions. Key strategies include dose adjustment, antibiotic de-escalation (which involves narrowing the spectrum of an antimicrobial agent after initial empirical treatment when culture and susceptibility data become available), and optimizing the timing and duration of antibiotic prophylaxis.

De-escalation, a cornerstone of ASP strategies, involves narrowing the spectrum of an antimicrobial agent after initial empirical treatment once culture and susceptibility data are available. A narrative review focusing on carbapenem de-escalation — a strategy aimed at minimizing unnecessary exposure to these broad-spectrum agents — found that such efforts reduced carbapenem use by 2 to 5 days (Gardner et al., 2025). The review concluded that de-escalation was not associated with negative clinical outcomes, such as higher rates of clinical failure or mortality, compared to the continuation of broad-spectrum therapy. While this evidence supports de-escalation as a promising strategy, the reviewed literature also highlights important nuances. Most studies are observational, and clinicians may be reluctant to de-escalate due to concerns about potentially compromising patient outcomes. This indicates that de-escalation must be approached on a patient-by-patient basis, guided by careful clinical judgment and supported by high-quality microbiology results. The evidence suggests that while ASPs provide a framework for these decisions, the ultimate success of de-escalation hinges on a collaborative, interdisciplinary approach and the ability of the ASP team to provide timely, individualized recommendations.

Furthermore, the impact of ASPs extends beyond drug consumption to include critical patient-centered outcomes such as hospital length of stay (LOS), mortality, readmission rates, and the incidence of hospital-acquired infections (HAIs). While the evidence for some of these outcomes is mixed, there are clear and consistent findings for others. Regarding hospital LOS and mortality, the literature presents a varied picture. A systematic review noted that the majority of studies (85%) reported a reduction or no change in LOS following ASP implementation, with decreases ranging from 0 to 22 days (Nathwani et al., 2019). However, more than half of the measured outcomes (53%) did not show a statistically significant change, and a few studies even reported an increase in LOS (Nathwani et al., 2019). Similarly, the impact on mortality rates has been variable, with studies showing either a decrease or no significant difference (Gardner et al., 2025). This heterogeneity in clinical outcomes suggests that while ASPs contribute to improved patient care, the improvements are not always direct or universally significant, as these metrics are influenced by many factors outside the direct control of the ASP team.

In contrast, the evidence for the impact of ASPs on *Clostridioides difficile* infection (CDI) rates is remarkably strong and consistent. A systematic review and meta-analysis of 16 studies demonstrated a significant protective effect of ASPs, with a pooled risk ratio of 0.48, indicating a substantial reduction in CDI incidence (Feazel et al., 2014). The mechanism behind this benefit is a clear example of how ASP interventions lead to positive clinical outcomes. ASPs often focus on reducing the use of high-risk antibiotics, which are a major driver of CDI (Barker et al., 2017). By limiting exposure to these agents through restrictive policies or prior approval requirements, ASPs directly contribute to a reduction in CDI, which in turn can lead to shorter hospital stays and improved patient safety. This interconnectedness highlights a crucial point: the benefits of ASPs often occur as a cascade of effects, where an improvement in one area (e.g., prescribing practices) leads to a positive outcome in another (e.g., reduced infection rates). Furthermore, the literature emphasizes that ASPs and Infection Prevention and Control (IPC) are complementary disciplines (Morrill et al., 2016). The integration of these two programs yields the best results, as IPC aims to prevent infections altogether, while ASPs work to ensure that the antibiotics prescribed are used with the highest quality and appropriateness. The reduction of infections, such as CDI and other HAIs, is a direct result of this synergistic relationship.

Studies have shown that ASPs are associated with a decrease in the prevalence of specific multidrug-resistant organisms such as Methicillin-Resistant *Staphylococcus aureus* (MRSA) and Vancomycin-Resistant *Enterococcus* (VRE) (Candra et al., 2024). The impact on bacterial susceptibility has also been documented. One retrospective analysis observed significant changes in susceptibility patterns following the implementation of an ASP, including an 8% increase in *Pseudomonas aeruginosa* susceptibility to tobramycin and a 7% increase in *Staphylococcus aureus* susceptibility to methicillin (Timbrook et al., 2016). However, the same study also reported a simultaneous decrease in susceptibility to another agent, piperacillin-tazobactam (Timbrook et al., 2016). This contradictory finding illustrates that the relationship between reduced antibiotic use and changes in resistance is complex and may not follow a simple linear cause-and-effect pattern. The effect can be differential, varying by bacterial species and antimicrobial class. This complexity underscores the critical role of surveillance and data-driven strategies in the long-term success of an ASP.

ASP teams must also work closely with clinical microbiology laboratories. Data on local resistance rates are essential for ASP teams to assess the existing burden of AMR and to make informed decisions about which antimicrobial agents to target for restriction or optimization (Khadse et al., 2023). The ability to continuously monitor trends and adapt stewardship policies is what enables a program to effectively combat evolving resistance patterns. Without this dynamic feedback loop, a program’s impact on resistance could be short-lived or inconsistent. The integration of continuous surveillance and data-driven interventions transforms an ASP from a static set of rules into a responsive and effective program that can influence the complex landscape of microbial resistance.

A systematic review of 27 studies on the economic impact of ASPs consistently demonstrated substantial cost savings. The findings revealed savings in antibiotic costs ranging from 2% to 95%, reductions in length-of-stay costs from 3% to 85% and decreases in overall hospital costs from 3% to 86% (Huser et al., 2025). In the United States, average cost savings of $732 per patient were reported, with a wide range from $2.50 to $2,640 (Nathwani et al., 2019). A critical finding from this analysis is that the primary driver of these overall cost reductions is not merely the savings on antibiotic procurement but rather the reduction in hospital length of stay.

Despite the above compelling evidence, the literature also reveals significant challenges in uniformly quantifying the economic benefits of ASPs (Antimicrobial Stewardship Programs, 2025). Studies on economic outcomes are often described as “sparse” and heterogeneous, making direct comparisons difficult (Antimicrobial Stewardship Programs, 2025). A more comprehensive economic analysis, as described in a recent study, distinguishes among three types of costs: “cost savings” (reduced drug costs), “cost avoidance” (costs prevented by avoiding adverse events such as readmissions or CDI), and “operational costs” (the cost of running the program) (Abushanab et al., 2024). The analysis highlights that while an ASP may achieve cost savings, cost avoidance can sometimes be negative, and the total operational costs may outweigh the direct savings. This indicates that a simple calculation of drug-cost reduction is insufficient to capture the full economic value of an ASP. A complete economic evaluation must account for all these variables, including the long-term financial benefits of preventing future infections and complications, to provide a more accurate and persuasive argument for continued investment in these programs (Abushanab et al., 2024).

All the evidence described above consistently supports the assertion that ASPs lead to more judicious use of antimicrobials and are associated with a range of positive benefits for patient care in inpatient settings. However, the current body of literature is not without its limitations. The primary challenge is the predominance of observational and quasi-experimental studies, which, while valuable, make it difficult to establish a definitive causal relationship between ASP interventions and observed outcomes. There is also significant heterogeneity in the specific interventions and the metrics used to measure their effectiveness, which hampers the ability to compare results across studies and institutions. Economic evaluations, in particular, are sparse and often fail to account for the full spectrum of costs, including the operational costs of the program and the long-term benefits of cost avoidance.

To address these limitations, future research should focus on conducting more high-quality, prospective interventional studies and randomized controlled trials. There is also a pressing need for the field to develop and adopt standardized metrics for both clinical and economic outcomes to improve the comparability and generalizability of research findings. Such standardization would enable a more accurate assessment of the benefits of ASPs and provide a stronger evidence base for justifying their implementation and expansion. Despite the challenges in measurement and the acknowledged heterogeneity of the evidence, the established benefits of ASPs in combating the antimicrobial resistance crisis affirm their essential and indispensable role as a cornerstone of modern public health.

### A framework for strengthening AMR surveillance systems in Africa

**Figure 1:**
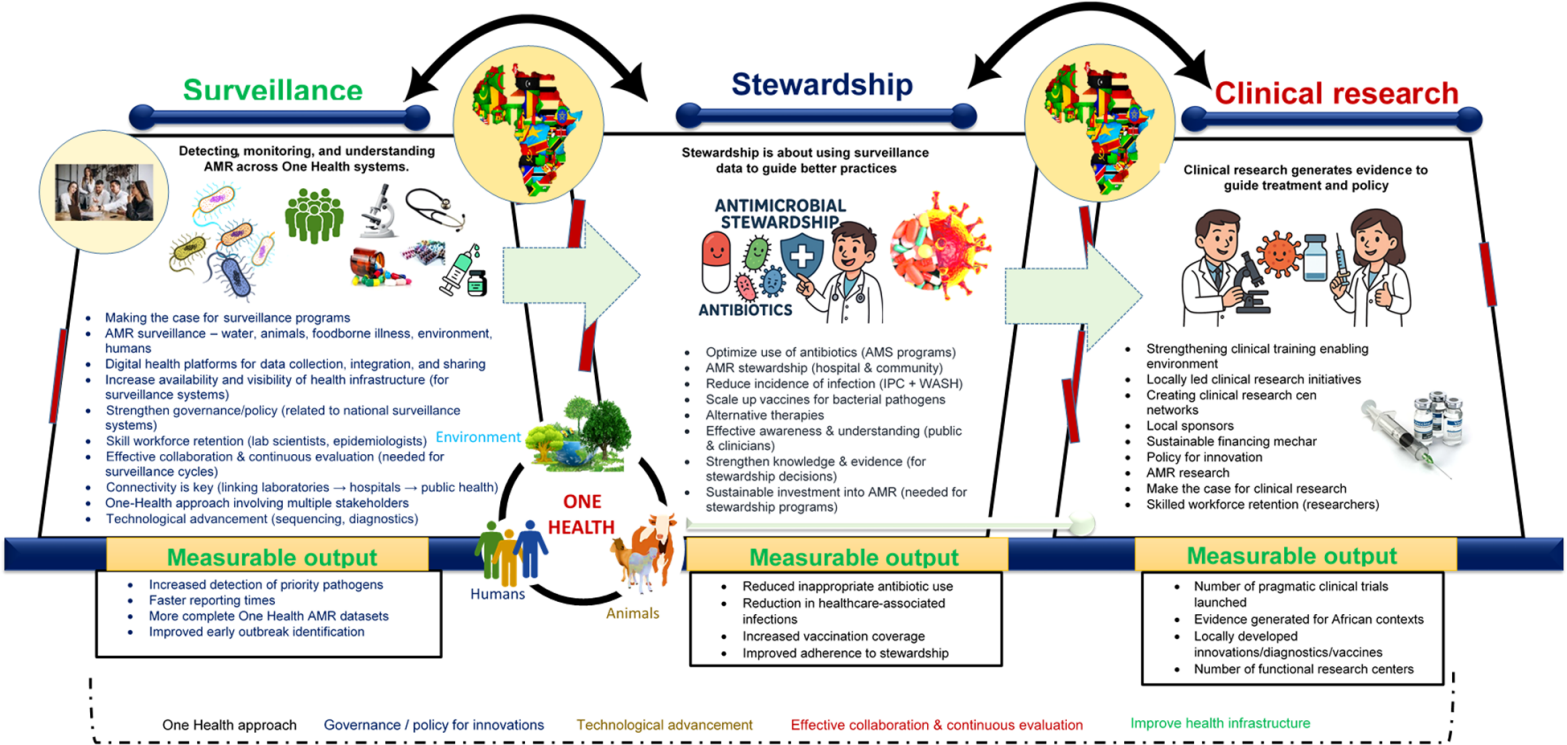
Conceptual framework illustrating the three core pillars required to build an effective AMR response system—Surveillance, Stewardship, and Clinical Research—and the cross-cutting enablers needed to link them. The framework is organized into three stacked layers showing the flow from Surveillance (AMR data collection across One Health sectors) to Stewardship (evidence-informed actions to optimize antibiotic use and reduce infections), and finally to Clinical Research (generation of locally relevant evidence and innovations). Arrows indicate how outputs from each layer feed into the next. Cross-cutting elements—including governance, policy, technology, connectivity, collaboration, and the One Health approach—span all layers and support system functionality. Any intervention aimed at improving public health must involve all the included critical components. However, the process must begin by clearly identifying the issues and making a compelling case for why specific interventions are necessary. Ultimately, connectivity is essential. All stakeholders - including government agencies, clinicians, pharmaceutical companies, international health experts, and patients - have vital roles to play, and these roles must be clearly defined. Progress depends on effective collaboration, continuous evaluation, and the open sharing of lessons learned. Most importantly, the One Health approach must be embraced to ensure a holistic and sustainable impact on health systems. Acronyms used in the figure: AMR, antimicrobial resistance; AMS, antimicrobial stewardship; IPC, infection prevention and control; WGS, whole-genome sequencing; LMIC, low- and middle-income countries.

To reduce the burden of AMR in Africa, it is essential to design an equitable, context-specific, and multidimensional framework. This process must begin with a clear understanding of on-the-ground realities rather than relying solely on externally developed interventions. Central to this approach is the establishment of robust surveillance systems. Without a clear picture of where resistance is emerging and how it is spreading, interventions will remain generalized and ineffective. Surveillance provides essential context, reveals critical gaps, and guides actionable, evidence-based strategies. Strengthening surveillance systems is key to generating high-quality data that support precision healthcare, inform targeted treatment protocols and vaccine development, and enhance our understanding of the epidemiology of resistant pathogens (Bertagnolio et al., 2023) (**Figure 1**).

Enhanced surveillance also enables the identification and tracking of important bacterial clones circulating across African regions and can uncover novel or emerging resistance mechanisms. However, this is only possible when both phenotypic and patient-related data are rigorously collected, standardized, and quality assured. A notable example is the study by Liu et al. (2016), which, through routine surveillance, identified the mcr-1 gene — a plasmid-mediated resistance mechanism to colistin — in *Escherichia coli* isolates from animals, meat, and humans in China. This discovery sparked global concern over the potential spread of untreatable multidrug-resistant infections. Similarly, a European surveillance study reported the emergence and dissemination of the *E. coli* ST131 clone carrying carbapenemase genes (Kohlenberg et al., 2024). These examples underscore the power of genomic surveillance in detecting resistance trends, shaping effective interventions, and highlighting the need for genomic tools in addressing health-related challenges.

Surveillance has also proven crucial in identifying AMR-related neonatal sepsis. For instance, a study by Sands et al. (2021) linked high neonatal mortality to sepsis caused by drug-resistant pathogens. Alarmingly, in many of these cases, the pathogens were either not detected or were identified too late for effective treatment. This further demonstrates the importance of appropriate and timely diagnosis, which relies heavily on genomics and its potential to influence patient outcomes.

While AMR surveillance is critical to addressing these challenges, surveillance efforts in Africa face numerous obstacles. These include weak laboratory infrastructure, limited access to consumables, diagnostics, and reagents, supply chain constraints, shortages of trained personnel, and insufficient funding (Iskandar et al., 2021; Mba et al., 2023). This raises an important question: how do we move forward? The answer lies in designing a framework that is both practical and scalable. First, there is a need to invest in sustainable surveillance infrastructure, which is essential not only for AMR control but also for strengthening other areas of public health that are vital for securing future generations. Second, core public health systems must be reinforced to support ongoing surveillance efforts and ensure their sustainability. Third, functional reference laboratories and strong regional networks must be established, as they are critical for promoting data interoperability and facilitating timely cross-border data sharing. In addition, genomic tools must be integrated into routine surveillance systems at all levels. While these tools may be costly to implement, the wealth of information derived from them is invaluable (Ndembi et al., 2024).

Furthermore, quality assurance is essential to ensure that surveillance data are accurate and comparable across sites. Establishing national quality control programs, conducting regular proficiency testing, and implementing clear, step-by-step procedures for sample collection, testing, and data entry all help ensure reliable results. Conducting studies, clinical trials, and implementation research at key surveillance sites can reveal how treatments perform, what drives resistance locally, and how effective stewardship programs are. Integrating data from hospitals, animal health services, agriculture, and environmental monitoring provides a more comprehensive view of how resistance spreads. While emphasis has been placed on generating quality data, it is also important to note that robust digital data management systems are vital and indispensable, as they unify disparate data streams and facilitate real-time reporting. Moreover, sustainable funding and strong partnerships are crucial for maintaining AMR surveillance systems over time. Governments across Africa should allocate domestic resources to reduce dependence on external support and work closely with partners — including the private sector, charities, and development organizations — to ensure adequate and sustained funding.

Finally, it is important to emphasize that surveillance should not be a reactive measure triggered only by crises. To fully harness its potential, it must be an active, continuous process that informs precision healthcare, guides antimicrobial stewardship, and supports vaccine development.

### A framework for strengthening clinical research in Africa

Medical interventions and public health measures are direct or indirect outputs of clinical research. However, to date, there remains a significant gap in clinical research in Africa — a continent where AMR is also deeply embedded. As previously mentioned, only 2-4% of global clinical trials are conducted in African countries and include African participants (Kizub et al., 2022; Ndembi et al., 2024). Currently, only four countries in Africa are committing and spending up to 15% of their GDP on health and science, as declared by WHO in 2001 (reference). In fact, the return on investment in science research and community health is estimated to be nearly 20 to 1 (Thusini et al., 2022). It is within our political and institutional capacity to invest in and conduct clinical trials in Africa. However, there is a pressing need to first strengthen health systems so that they can deliver care in a consistent and reproducible manner. Only then will it be possible — and easier — to integrate clinical research and trials into local healthcare facilities.

Transforming Africa’s research ecosystem is not optional — it is imperative. This transformation involves reimagining and redesigning systems essential for high-quality healthcare delivery and scientific productivity, while creating a favorable environment for clinical research and trials. While Africa has the political will to invest in and conduct such research, the foundational step must be to strengthen health systems so they can reliably deliver quality care. Only then can clinical research be effectively embedded into local healthcare settings. The role of technology in this transformation cannot be overstated (Segun-Omosehin et al., 2025). Looking ahead, building resilient health systems capable of consistently delivering care — and embedding research into routine practice — is essential.

A coordinated and integrated clinical research ecosystem is urgently needed: one that overcomes structural limitations, secures sustainable financing, fosters innovation, and supports trials addressing local health priorities. Investing in clinical research is not only a scientific necessity but also an economic opportunity. Every dollar spent can yield substantial returns by enabling health interventions tailored to the unique genetic, cultural, and environmental contexts of African populations. Africa must also transition from fragmented, short-term projects to a cohesive and practical research framework that delivers tangible health benefits. The continent must move away from crisis-driven responses toward proactive, long-term strategies. This transformation requires a thorough understanding of structural barriers, the development of sustainable funding models, and the creation of an enabling environment for long-term scientific engagement. Without strategic investment in clinical research, Africa risks continued exclusion from global innovations that are critical for improving public health outcomes.

Integrating clinical research within sentinel surveillance site activities will also accelerate the translation of data into actionable interventions. For example, linking hospital-based surveillance with antimicrobial use monitoring can inform guideline development and stewardship training in African nations. Finally, expanding research and development (R&D) across different sectors and leveraging innovations — such as genomic sequencing technologies developed during the COVID-19 pandemic — will make a significant difference. Overall, with coordinated action and strong political commitment, Africa can emerge as a global leader in health innovation and secure a healthier, research-driven future (**Figure 1**).

### Closing the research and development gap and addressing the mundane things

Doing real science in Africa is possible — but in practice, it remains extremely challenging. To make scientific progress more accessible and impactful, there must be sustained commitment to building a robust framework, a supportive ecosystem, and long-term partnerships rooted in trust and investment. While funding is often emphasized, several overlooked yet critical barriers continue to hinder scientific advancement in low- and middle-income countries, particularly in Africa. Unless these issues are addressed, the full potential of scientific and health initiatives will remain unrealized. Many of these barriers involve practical, day-to-day challenges that researchers in high-income countries rarely face.

One of the most pressing issues is supply chain inefficiency (Kamara and Essien, 2022; Klibi et al., 2025). When essential consumables and diagnostic equipment cannot be imported or distributed efficiently, it becomes nearly impossible to deliver timely care or conduct meaningful research. The consequences are severe: prolonged hospital stays, delayed outbreak responses, and increased transmission risk — often due to persistent infections that could otherwise be managed or contained. If consumables and equipment needed for diagnosis and research are not available, it becomes a major barrier to public health. The situation is worse than many might imagine. Currently, the turnaround time for most outbreak cases in a country like Nigeria can be several weeks, months, or even years in some instances, due to the unavailability of materials needed to verify cases and provide immediate solutions or treatment. Sadly, most of these outbreaks are caused by multidrug-resistant pathogens. When these pathogens are not contained, they spread to others, further complicating the situation. In many cases, outbreak data become available only when the outbreak is already over — by which time patients may have recovered, died and been buried, or remained hospitalized for extended periods due to persistent infections.

Another major challenge is talent retention (Talent Retention, 2025). Africa is home to many brilliant scientific minds, yet a significant number leave the continent in search of better opportunities. While global mobility can foster career development and knowledge exchange, there is an urgent need for bidirectional movement. Incentivizing African scientists abroad to return and contribute to local innovation ecosystems is also crucial. Establishing a stable, well-funded research environment with long-term institutional support and trustworthy partnerships will help retain talent and reverse the ongoing brain drain. A thriving research ecosystem not only fosters innovation but also strengthens community health by building local capacity to address pressing health challenges.

In the agricultural sector, a striking imbalance exists: marketing strategies often outpace investment in R&D (El Mahdy, 2021; Abate et al., 2023). Many farmers prioritize profit over safety by aggressively promoting practices that deliver short-term gains (e.g., faster animal growth using antibiotics) at the expense of long-term sustainability and public health. Many also rely on outdated practices, including the misuse of antibiotics for growth promotion and disease prevention, which contributes to the spread of AMR (Hedman et al., 2020; Zhang et al., 2024; Trinchera et al., 2025). Some continue to use outdated or unproven methods that pose risks to the ecosystem and indirectly or directly affect human health. Most of these problems stem from the fact that many farmers have access to marketing support but lack access to R&D expertise. This skewed focus promotes short-term, profit-driven practices that can harm public health — for example, through the overuse of antibiotics. Integrating agricultural R&D would encourage evidence-based practices, enhance sustainability, and reduce inappropriate antimicrobial use.

In the area of vaccine production, the situation is even more alarming. The COVID-19 pandemic exposed the region’s vulnerability, as 99% of vaccines used in Africa were imported (Ekström et al., 2021). This dependency revealed deep gaps in local R&D and manufacturing infrastructure. Encouragingly, scientists in South Africa have demonstrated that vaccine production on the continent is indeed possible (Lamptey et al., 2022). However, greater investment and international collaboration are needed to expand vaccine research and manufacturing capacity. Doing so would not only reduce the burden of drug-resistant infections but also address vaccine hesitancy by fostering local trust, ownership, and accessibility (Mba et al., 2023; Iwu-Jaja et al., 2025).

Furthermore, Africa has demonstrated its ability to rapidly mobilize in times of crisis. The establishment of the Africa Centres for Disease Control and Prevention (Africa CDC) following the West African Ebola outbreak is a prime example of this adaptive capability (Onyekuru et al., 2023). Since then, many countries have developed or strengthened their national public health institutions. Similarly, the COVID-19 pandemic accelerated the establishment of genomic sequencing capacity in several African countries (Mboowa et al., 2024). Today, there is a growing call to repurpose pandemic-era infrastructure, including sequencing platforms, for broader health applications. These developments demonstrate that effective systems can be built quickly in response to urgent needs.

However, rather than continually operating in crisis mode, Africa must shift towards proactive, long-term health strategies. Leveraging existing institutions such as Africa CDC and repurposing pandemic-era infrastructure can lay the foundation for sustainable, locally driven research. Real progress will not come from copying foreign models but from creating context-appropriate frameworks rooted in African realities. With coordinated efforts, political will, and sustained investment, Africa can transition from being a passive recipient of global health interventions to a leader in innovation, research, and public health resilience.

Therefore, establishing a stable and sustainable framework for genomic surveillance and clinical research—free from the pressures of emergency response—would enable better planning, consistent quality, and more impactful outcomes. Gaps in health infrastructure must be closed at all costs. In fact, the COVID-19 pandemic revealed significant data gaps in sub-Saharan Africa’s health systems. Information on facility capacity, availability of specialists, bed counts, and proximity to populations was often unavailable or unreliable. These data are essential for assessing hospital preparedness and ensuring equitable, timely access to care. Also, pragmatic trial designs are essential for generating locally relevant evidence that can be implemented within routine African health systems. The clinical-research pillar therefore emphasizes trial archetypes that are feasible, cost-efficient, and aligned with existing care pathways. **Table 1** outlines five examples of pragmatic trial designs appropriate for AMR-related interventions in African settings, including suggested sample units and measurable primary outcomes.

**Table 1:** Pragmatic trial archetypes suited to african health system contexts.

Trial archetype	Description	Sample unit	Primary outcome (Pragmatic)
1. Stepped-wedge IPC bundle trial	Sequential roll-out of IPC package (hand hygiene, cleaning, WASH improvements) across facilities.	Hospital ward or health facility (cluster)	Healthcare-associated infection rate per 1,000 patient-days.
2. Cluster randomized ASP trial	Facility-level antimicrobial stewardship program with training and audit-and-feedback vs usual care.	Hospital ward or full facility (cluster)	Proportion of antibiotic prescriptions that are guideline-concordant; or DOT per 100 patient-days.
3. Diagnostic stewardship/POC testing trial	Introduction of rapid diagnostics (CRP, antigen tests) + decision support to reduce unnecessary antibiotics.	Primary care clinic or outpatient department (cluster)	Proportion of febrile/respiratory patients receiving antibiotics within 7 days.
4. Hybrid effectiveness–implementation vaccine or prophylaxis trial	Tests both how well the vaccine/prophylaxis works *and* how well delivery strategies (decentralized cold chain, community mobilization) perform.	District, health-facility network, or community clusters	(Effectiveness) Incidence of target bacterial infection; (Implementation) Coverage rate or timeliness.
5. Adaptive platform trial for simplified antibiotic regimens	Multi-arm design comparing shorter or alternative oral antibiotic regimens using routine care pathways.	Individual patient or facility cluster	Clinical failure at 14–30 days (retreatment, hospitalization, or infection-related complications).

In summary, any system designed to support functional surveillance and clinical research in Africa must therefore incorporate all the components already discussed (**Figure 1**) and without reliable data systems, optimal community health outcomes will remain out of reach. When these elements are in place, Africa can evolve from a passive recipient of global health solutions into a global leader in innovation, resilience, and research-driven public health. By building context-specific frameworks grounded in African realities—and supported by coordinated action and strong political commitment—the continent can generate high-quality data, shape evidence-based policies, and improve health outcomes for future generations. By integrating strong governance, One Health approaches, laboratory strengthening, digital systems, quality assurance, embedded clinical research, sustainable financing, workforce development, and addressing the other issues mentioned, Africa can establish a functional AMR surveillance and clinical research framework that generates robust data, informs policy, improves patient outcomes, and prevents infectious diseases across the continent.

### Addressing the socio-political determinants shaping AMR prevention, surveillance, and stewardship

AMR prevention, surveillance, and stewardship do not operate in isolation; instead, they are embedded within complex governance structures, cultural norms, and resource limitations that significantly influence their effectiveness. One of the central challenges is the variability in political commitment and governance capacity across countries. In many settings, AMR competes with multiple pressing national priorities, and this competition can delay policy execution, weaken regulatory enforcement, and limit funding for surveillance infrastructure. Weak governance, combined with fragmented coordination across human health, veterinary, and environmental sectors, creates gaps that undermine both stewardship and infection-prevention efforts. Strengthening governance frameworks, ensuring inter-ministerial collaboration, and building accountability mechanisms are therefore essential components of any sustainable AMR strategy.

Economic constraints also play a major role. Many health facilities operate under chronic resource shortages, which affect everything from laboratory diagnostics to basic infection-control materials. These limitations frequently push clinicians toward broad-spectrum empirical prescribing and reduce the feasibility of data-driven stewardship. Even when advanced genomic or digital surveillance tools are available, the long-term costs of maintenance, consumables, and training may exceed local budgets. These realities underscore the need for balanced strategies that pair high-technology innovations with low-cost, immediately implementable interventions such as improved IPC practices, community education, and medicine regulation.

Cultural and community-level dynamics further shape antibiotic use. Self-medication, over-the-counter access to antibiotics, and reliance on informal providers are common in many regions and can undermine stewardship policies implemented at the national level. These behaviors are often driven by trust, affordability, and convenience rather than misuse alone. Without meaningful community engagement and culturally informed behavior-change strategies, the uptake of stewardship and surveillance programs may remain limited.

Finally, broader socio-political factors such as insecurity, population displacement, weak regulatory environments, and the presence of informal pharmaceutical markets complicate AMR control efforts. Conflict and instability disrupt surveillance networks and increase infection risks, while poorly regulated drug markets contribute to the circulation of falsified or substandard antibiotics. These contextual challenges reinforce the need for AMR policies that are resilient, adaptable, and rooted in an understanding of local realities. Overall, incorporating these socio-political considerations into AMR planning strengthens the feasibility and relevance of proposed interventions. A nuanced appreciation of governance dynamics, cultural practices, and economic pressures enables the design of strategies that are not only technically sound but also contextually grounded, equitable, and more likely to achieve long-term impact.

## The way forward and conclusion

First, the well-established benefits of ASPs in combating the AMR crisis, as demonstrated in many countries, affirm their essential and indispensable role as a cornerstone of modern public health. Therefore, ASPs need to receive greater attention in African settings. Although efforts have been made in most regions, several barriers continue to hinder the successful adoption of ASPs and infection prevention and control (IPC) programs across the continent (Harun et al., 2024).

Generally, across African settings, physicians have a suboptimal level of familiarity and knowledge regarding ASPs, and this area requires urgent attention. The lack of funding is also a pervasive challenge, affecting the development of infrastructure, the acquisition of essential resources, training, and the execution of monitoring operations. Inconsistent political commitment and weak governance structures further contribute to fragmented policies, poor enforcement of regulations, and insufficient cumulative investment in AMR control efforts. Moreover, the high prevalence of substandard and low-quality antibiotics in circulation undermines the effectiveness of treatment (Ndaki et al., 2025). Therefore, efforts must be intensified to address these challenges.

Governance is a key issue that shouldn’t be overlooked. The successful implementation of the Surveillance, Stewardship, and Clinical Research framework in African contexts depends heavily on effective governance. Complexities such as corruption, limited regulatory capacity, and competing health priorities can affect each layer: for example, weak oversight may compromise the quality and reliability of surveillance data, reduce adherence to stewardship interventions, and slow the establishment of locally led clinical research networks. Anticipating these challenges and integrating strategies for transparency, accountability, and cross-sector collaboration is essential to ensure realistic timelines and sustainable policy development. Addressing governance hurdles proactively strengthens the likelihood that AMR initiatives will be both effective and enduring.

While advanced genomic and digital surveillance tools offer powerful capabilities for detecting and tracking AMR, their implementation in resource-constrained African settings may face feasibility, cost, and sustainability challenges. In the short term, low-cost, high-impact interventions, such as public awareness campaigns, enforcement of existing regulations, and strengthening basic infection prevention and control measures—may provide more immediate benefits. A pragmatic approach that balances investment in advanced technologies with accessible interventions ensures both rapid impact and the gradual integration of high-tech solutions as capacity and funding allow, enhancing the overall effectiveness and applicability of AMR strategies. Moving forward, there is a need to invest in primary healthcare infrastructure and ensure equitable access to quality medical care, reliable diagnostics, and affordable essential medicines. These measures will help reduce inappropriate self-medication and the unregulated sale of antibiotics.

It is also crucial to adopt strict interventions, such as establishing robust WASH (water, sanitation, and hygiene) programs within healthcare facilities and communities, to prevent the spread of infections and ultimately reduce disease burden — and consequently, the demand for antibiotics (Okesanya et al., 2024; Akinsulie et al., 2024). Expanding access to affordable, rapid diagnostic tools at all levels of the healthcare system — from community health centers to tertiary hospitals — is pivotal. Such diagnostics will improve diagnostic accuracy, minimize empirical prescribing, and enable the selection of the most appropriate antibiotics. Currently, hospital-acquired infections are common but often go undetected in many African hospitals, largely due to diagnostic gaps — an area that requires urgent attention.

There is also a need to establish and enforce policies on the manufacturing, importation, distribution, and sale of antimicrobials to combat substandard and falsified drugs. This should include robust post-market surveillance. Guidelines on the safe disposal of pharmaceutical waste must be developed, implemented, and enforced, while antibiotic pollutants in the environment should be closely monitored, particularly in wastewater and agricultural run-off (Ajekiigbe et al., 2025; El-Maradny et al., 2025). Laws restricting the indiscriminate sale of antibiotics—especially without a prescription—and awareness-raising programs on the risks of self-medication are also crucial (Kotwani et al., 2023). There is also a need to develop and implement regulations governing the use of antibiotics in livestock farming and agriculture that promote responsible use, including the withdrawal of antibiotics as growth promoters (Matheou et al., 2025; Kasimanickam et al., 2021; Trinchera et al., 2025). Establishing national coordination centers and AMR committees across all regions can facilitate policy development, ensure compliance with regional policies, enable data sharing, and improve resource distribution across the health sector. The Africa CDC’s AMRSNET program (Takah et al., 2017) is a good example of this approach, but a stronger and more effective system is still needed.

An important limitation of relying on national-level indicators of government effectiveness and health investment is that these aggregated measures can obscure substantial disparities within individual countries. In many African settings, health system capacity, access to diagnostics, and the burden of AMR vary widely between urban and rural areas, across different administrative regions, and among marginalized or underserved populations. These differences have direct implications for the implementation of surveillance systems, stewardship programs, and infection-prevention measures. For instance, tertiary hospitals in urban centers may have stronger laboratory capacity and antimicrobial oversight, while peripheral facilities may face persistent shortages of staff, supplies, or functioning infrastructure. Recognizing these inequities underscores the need for more disaggregated data—by region, facility type, and population group—to better capture localized vulnerabilities and tailor interventions accordingly. Incorporating this nuance strengthens the understanding of contextual barriers and enhances the relevance and equity of AMR control strategies.

Also, building effective AMR surveillance and trial systems requires robust and equitable data governance structures that protect privacy, promote fairness, and ensure responsible data use. African-led frameworks, such as the African Union Data Policy Framework and the Africa CDC Framework for Data Sharing, provide guidance on transparency, accountability, community engagement, and fair benefit sharing. Integrating these frameworks ensures that surveillance data and clinical research outputs support local priorities, strengthen health systems, and deliver equitable benefits to participating communities. Also strengthening AMR surveillance, stewardship, and clinical research systems in Africa requires acknowledging structural risks that can undermine long-term sustainability. Key challenges include supply-chain instability, loss of skilled personnel, and unpredictable funding flows. **Table 2** summarizes these risks and outlines practical mitigation strategies that align with regional priorities and existing African health system frameworks.

**Table 2:** Key risks to strengthening AMR surveillance and clinical research capacity and potential mitigation strategies.

Risk area	Description	Mitigation strategies
Supply-chain instability	Delays or shortages in diagnostics, laboratory consumables, sequencing reagents, and essential medicines.	Framework contracts with multiple vetted suppliers
• Regional pooled procurement mechanisms		
• Support for local manufacturing and stockpiling		
Talent retention challenges	Loss of skilled laboratory staff, data scientists, clinicians, and AMR researchers to better-funded institutions or other countries.	Diaspora sabbaticals and visiting-scientist schemes
• Competitive career pathways and protected research time		
• Regional centers of excellence for training		
Funding volatility	Short-term, unpredictable, or donor-dependent financing leading to interruptions in surveillance systems and trials.	Multi-year funding agreements
• Blended financing models and national co-funding		
• Integration of AMR activities into essential health budgets		

Strengthening governance for AMR control requires not only high-level commitment but also clear, actionable steps to guide policy reform and institutional development. Practical measures include establishing national AMR coordinating bodies with defined mandates, budget lines, and reporting structures to ensure accountability across sectors. Reinforcing regulatory frameworks—particularly around antimicrobial distribution, prescription practices, and quality control—can be achieved through periodic legislative review, stronger enforcement mechanisms, and collaboration with professional councils. Capacity-building within ministries of health, agriculture, and environment is equally critical and may involve training programs in policy analysis, data governance, and cross-sectoral coordination. At the operational level, countries can adopt standardized surveillance guidelines, integrate AMR indicators into existing health information systems, and develop institutional performance dashboards to monitor progress. Investing in governance also requires mechanisms for transparency and community participation, such as public reporting of AMR trends, stakeholder consultations, and civil society engagement in oversight processes.

Antimicrobial stewardship and infection control programs must also be introduced in every healthcare facility—starting from primary care centers to tertiary hospitals—as a key component of national health policies. Before the implementation of these programs, clear directives on how they should be carried out must be established, along with a plan for regular monitoring and evaluation. Physicians, pharmacists, nurses, microbiologists, community health workers, and local leaders should all be involved in ensuring effective implementation and raising awareness about appropriate antibiotic use and hygiene practices. Every African nation must also invest in surveillance systems that generate the evidence needed to monitor AMR. Because various behavioral, social, and cultural factors influence AMR in Africa, these factors must be taken into account to collect comprehensive data that can inform context-specific interventions (Kapatsa et al., 2025; Bennett et al., 2024; Lubanga et al., 2025). Moreover, regional and international cooperation in knowledge sharing, capacity building, and resource mobilization must be strengthened. The One Health concept must also be fully embraced, as it is central to addressing AMR in Africa. A One Health approach is essential for capturing the full spectrum of AMR emergence and transmission. When supported by strong political will and sustained commitment, it offers a clear pathway to containing AMR and safeguarding Africa’s future. Additionally, efforts and research into alternative antibiotic therapies must be intensified.

In summary, AMR poses a serious threat to health, food security, and economic stability in Africa, requiring urgent and coordinated action. While this review has outlined key recommendations, the most critical step remains the adoption of evidence-based strategies tailored to Africa’s unique realities. Strengthening surveillance, improving stewardship, investing in affordable diagnostics, and fostering cross-sector collaboration are essential. To effectively mitigate antimicrobial resistance, policymakers, healthcare practitioners, and global stakeholders must recognize the urgent need for timely, evidence-based, and context-specific interventions. Immediate action is imperative, as further delays will only worsen the already severe public health burden and economic consequences associated with resistance.
